# Up-regulation of CTD-2547G23.4 in hepatocellular carcinoma tissues and its prospective molecular regulatory mechanism: a novel qRT-PCR and bioinformatics analysis study

**DOI:** 10.1186/s12935-018-0566-3

**Published:** 2018-05-11

**Authors:** Dong-yue Wen, Peng Lin, Hai-wei Liang, Xia Yang, Hai-yuan Li, Yun He, Hong Yang, Gang Chen

**Affiliations:** 1 Department of Medical Ultrasonics, First Affiliated Hospital of Guangxi Medical University, Nanning, 530021 Guangxi Zhuang Autonomous Region People’s Republic of China; 2 Department of Pathology, First Affiliated Hospital of Guangxi Medical University, Nanning, 530021 Guangxi Zhuang Autonomous Region People’s Republic of China

**Keywords:** CTD-2547G23.4, Hepatocellular carcinoma (HCC), Bioinformatic analysis, qRT-PCR, TCGA, GEO

## Abstract

**Background:**

Dysregulated expression of long non-coding RNAs (lncRNAs) has been reported in the pathogenesis and progression of multiple cancers, including hepatocellular carcinoma (HCC). LncRNA CTD-2547G23.4 is a novel lncRNA, and its role in HCC is still unknown. Here, we aimed to clarify the expression pattern and clinical value of CTD-2547G23.4 and to investigate the prospective regulatory mechanism via bioinformatics analysis in HCC.

**Methods:**

To identify differentially expressed lncRNAs in HCC, we downloaded RNA-Seq data for HCC and adjacent non-tumour tissues via The Cancer Genome Atlas (TCGA). CTD-2547G23.4 was selected by using the R language and receiver operating characteristic curve analysis. Furthermore, we validated the differential expression of CTD-2547G23.4 via Gene Expression Omnibus (GEO), ArrayExpress, Oncomine databases and quantitative real-time polymerase chain reaction (qRT-PCR). The relationship between the CTD-2547G23.4 level and clinic pathological parameters was also assessed. To further probe the role of CTD-2547G23.4 in HCC cell cycle, lentivirus-mediated small interfering RNA was applied to silence CTD-2547G23.4 expression in Huh-7 cell line. In addition, the related genes of CTD-2547G23.4 gathered from The Atlas of Noncoding RNAs in Cancer (TANRIC) database and Multi Experiment Matrix (MEM) were assessed with Gene Ontology (GO), Kyoto Encyclopedia of Genes and Genomes, Protein Analysis Through Evolutionary Relationships and protein–protein interaction (PPI) networks.

**Results:**

CTD-2547G23.4 expression was remarkably higher in 370 HCC tissue samples than that in adjacent non-tumour liver tissues (48.762 ± 27.270 vs. 14.511 ± 8.341, P < 0.001) from TCGA dataset. The relative expression level of CTD-2547G23.4 in HCC was consistently higher than that in adjacent non-cancerous tissues (2.464 ± 0.833 vs. 1.813 ± 0.784, P = 0.001) as assessed by real time RT-qPCR. The area under the curve of the summary receiver operating characteristic curve was 0.8720 based on TCGA, qRT-PCR and GEO data. Further analysis indicated that the increased expression levels of CTD-2547G23.4 were associated with the neoplasm histologic grade and vascular tumour cell type. The expression of CTD-2547G23.4 was significantly downregulated in CTD-2547G23.4 knockdown cells. Moreover, cell cycle analysis revealed that CTD-2547G23.4 depletion in Huh-7 cell line led to S phase arrest. Furthermore, 314 related genes identified by TANRIC and MEM databases were processed with a pathway analysis. The bioinformatics analysis indicated that CTD-2547G23.4 might play a key role in the progress of HCC through four hub genes, SRC, CREBBP, ADCY8 and PPARA.

**Conclusions:**

Collectively, we put forward the hypothesis that the novel lncRNA CTD-2547G23.4 may act as an exceptional clinical index and promote the HCC tumourigenesis and progression via various related genes.

**Electronic supplementary material:**

The online version of this article (10.1186/s12935-018-0566-3) contains supplementary material, which is available to authorized users.

## Background

Hepatocellular carcinoma (HCC) is currently a highly prevalent human cancer, which is correlated with high mortality all over the world [1]. Moreover, the number of new cases of HCC is increasing with each passing year. The number of HCC patients in the United States is predicted to reach approximately 27,000 by 2020 [2]. The risk factors of HCC primarily include cirrhotic livers and chronic liver injury caused by virus infections [3]. Although developments in the screening and treatment of HCC have been rapid, the clinical outcome is still limited due to frequent recurrence and metastasis regulated by the activation of multiple signal transduction pathways [4–6]. Prior to our study, extensive research has focused on new biomarkers associated with HCC diagnosis, prognosis, and evaluation of treatment efficacy [7–11]. However, satisfactory biomarkers and therapeutic related genes of HCC are still rare and sought-after. Therefore, the identification of a novel reliable biomarker and further investigation of the molecular mechanisms for HCC, which could boost the diagnostic value and survival prediction, are imperative.

Long noncoding RNAs (lncRNAs) are transcriptional RNA molecules with little protein-coding capacity that are longer than 200 nucleotides [12]. Lately, new lncRNAs have been extensively discovered. Fortunately, studies on lncRNAs and their roles in various pathophysiological processes have created new avenues for cancer diagnosis and therapies [13]. LncRNA SPRY4-IT1 has been shown to promote proliferation and invasion of HCC through activating EZH2 and may be used as a new treatment biomarker [14]. Furthermore, Lv et al. [15] reported that lncRNA Unigene56159, as a ceRNA of miR-140-5p, thus promotes the migration and invasion of HCC cells. Despite the fact that several lncRNAs have been demonstrated to be indispensable in the biological process of HCC, screening novel lncRNAs which could be excellent diagnostic and prognostic markers are still urgently needed.

For the sake of exploring the novel lncRNAs which could serve as an appropriate clinical index and investigate the potential mechanisms responsible for HCC, CTD-2547G23.4 (ENSG00000274925, Exons: 1, Coding exons: 0, Transcript length: 3115 bps), a lncRNA, has not yet been explored to play a potential role in the molecular mechanism of cancers, was finally randomly chosen due to its good diagnostic value and its unknown biological function. Here, we proposed to uncover the mystery of CTD-2547G23.4.

## Materials and methods

### TCGA dataset and analysis of the differentially expressed lncRNAs

TCGA project provides RNA sequencing (RNA-Seq) data from 370 HCC cases and 50 adjacent liver tissue samples. The publicly available RNA-Seq data were downloaded directly from the TCGA portal (https://cancergenome.nih.gov/) via bulk download mode of the liver hepatocellular carcinoma (LIHC) (cancer type), RNASeqV2 (data type), and level 3 (data level) cancer tissues collected by the end of December 8, 2016. The lncRNA expression data were displayed as HTSeq-Counts. Next, analysis was carried out using the DESeq package in R language to compare the lncRNA expression data from HCC and their adjacent non-tumour tissues. Differentially expressed lncRNAs between HCC tissues and the adjacent non-tumour tissues were selected based on the following criteria: Padj < 0.05 and an absolute log_2_FC > 1. Visualization of the identified differentially expressed lncRNAs is shown in the form of a volcano plot that was created by using the ggplot2 package. Receiver operating characteristic (ROC) curve analyses were performed to identify the distinguishing capability of cancer from non-cancerous tissues. Based on the AUC values, the lncRNA CTD-2547G23.4, was finally selected for in-depth investigation.

### Verification of CTD-2547G23.4 expression based on other databases

We also collected RNA-Seq or chip datasets from Gene Expression Omnibus (GEO) (https://www.ncbi.nlm.nih.gov/geo/), ArrayExpress (https://www.ebi.ac.uk/arrayexpress/) and Oncomine (https://www.oncomine.org/resource/login.html) databases. The following keywords were used: (“lncRNA” OR “lncRNAs”) AND (malignan* OR cancer OR tumour OR tumour OR neoplas* OR carcinoma) AND (hepatocellular OR liver OR hepatic OR HCC). We extracted all of the expression data for CTD-2547G23.4 and the clinical parameters from the online public databases.

### Confirmation of CTD-2547G23.4 expression based on clinical samples

Hepatocellular carcinoma tissue samples and their adjacent normal liver tissues were gathered from 39 HCC patients from the First Affiliated Hospital of Guangxi Medical University, People’s Republic of China from January, 2012 to August, 2013. All formalin-fixed, paraffin-embedded (FFPE) clinical samples were acquired from the surgical resection of HCC patients who had not received tumour-specific therapy prior to surgery. The diagnoses were independently confirmed by two pathologists (Hai-wei Liang and Gang Chen), and the adjacent non-cancer hepatic tissues located at least 2 cm away from the macroscopically unaffected margins of the tumour were confirmed as being without cancer by microscopic analysis. The Ethics Committee of First Affiliated Hospital of Guangxi Medical University approved the protocol, and written informed consent was provided by HCC patients involved.

### RNA extraction and quantitative real-time PCR

Total RNA was extracted from 39 HCC samples and corresponding adjacent non-tumour tissues using the Qiagen RNeasy FFPE Kit following the manufacturer’s protocol as previously reported [16, 17]. The quantification of CTD-2547G23.4 and glyceraldehyde-3-phosphate dehydrogenase (GAPDH) was performed by Applied Biosystems PCR7900. The sequences of the CTD-2547G23.4 primer were as follows: F: TTTGGTCTCTTCGGGTCATC, R: CTTAGCTGGACGCCTACCTG. For GAPDH, the primers were: F: GTAAGACCCCTGGACCACCA, R: CAAGGGGTCTACATGG CAACT. The expression were calculated using the 2^−ΔCt^ method.

### Cell culture

Huh-7, SMMC-7721, HepG2, Bel-7404 and HL-7702 cell lines, were all purchased from the Cell Bank of Chinese Academy of Sciences. Five human HCC cell lines were cultured in Dulbecco’s modified Eagle’s medium (DMEM, HyClone, Logan, UT), mixed with 10% fetal bovine serum at 37 °C in the presence of 5% CO_2_.

### Lentivirus construction and transfection

To further probe the role of CTD-2547G23.4 in HCC cell cycle, lentivirus-mediated siRNA was applied to silence CTD-2547G23.4 expression in Huh-7 cell line. According to the CTD-2547G23.4 sequence from Ensemble (ENSG00000274925), three different target siRNA sequences were designed and named as knock down-1 (KD-1) (5′-CAGCCTTCATTCAGTGGCCAT-3′), KD-2 (5′-AGGCATTGACCAAGAAAGTAA-3′), KD-3 (5′-TCGCTTGAGGTCAGAAGTTTA-3′), with a negative control (NC) siRNA target sequence (5′-TTCTCCGAACGTGTCACGT-3′). Above-mentioned siRNA fragments were cloned into a GV248 vector (Shanghai GeneChem, China).

### Cell cycle analysis

The fluorescence intensity of propidium directly detected by flow cytometry, which reflected the distribution status of DNA in the G0/G1, S and G2/M phases. Cells were seeded in 6 cm dish at a density of 4 ml/well. According to the manufacturer’s protocol, cells with 80% confluence were stained with propidium after lentivirus infection for 5 days, and determined by a flow cytometer (Guava easyCyte HT, Millipore).

### Statistical analyses for the clinical implication of CTD-2547G23.4

Statistical analyses were performed utilizing the SPSS 24.0 statistical software package (Chicago, IL, USA), graphs and curves were constructed with the GraphPad Prism 7 software (GraphPad Software, San Diego, CA, USA). The quantitative values were expressed as the mean ± SD (range). Student’s t test was adopted for the comparison of two independent groups. We used ROC and summary ROC (SROC) curves to examine the feasibility of using the CTD-2547G23.4 expression level as a value for screening or detecting HCC. Then, the overall SMD with a 95% CI was assessed with STATA software version 12.0 (StataCorp, College Station, TX, USA). An observed SMD > 0 with a 95% CI not crossing zero indicated that CTD-2547G23.4 had a higher expression level in HCC tissues than in adjacent non-tumour tissues. The heterogeneity across datasets was analysed with the I^2^ statistics method. A P value less than 0.05 or an I^2^ more than 50% was considered to indicate a heterogeneous dataset, in which a random-effects model would be used for pooling data. Otherwise, a fixed-effects model was employed. All statistical tests were two-sided. The statistical results were considered to be significant with a P value less than 0.05.

### Prospective related genes of CTD-2547G23.4 in HCC

The related genes of CTD-2547G23.4 were accumulated based on two online prediction databases: TANRIC (http://ibl.mdanderson.org/tanric/_design/basic/index.html) and MEM (https://biit.cs.ut.ee/mem/) databases. TANRIC database is a web resource of the Bioinformatics and Computational Biology department used to explore the interactions of lncRNAs in cancer and comprises experimentally supported mRNA related genes [18]. MEM is a gene expression query and visualization tool from a web-based multi-experiment that gathers substantial publicly usable gene expression data from the ArrayExpress database [19]. To identify more reliable pathways and gene network, we examined the intersecting genes from the above two sets of TANRIC and MEM.

### Gene-enrichment and functional annotation analysis

To further understand the potential mechanism of CTD-2547G23.4 in HCC, the Database for Annotation, Visualization and Integrated Discovery (DAVID, https://david.ncifcrf.gov/) was used to perform a GO enrichment analysis, KEGG and PANTHER pathway annotations. GO terms, KEGG and PANTHER pathways with P < 0.05 were considered significant. The enrichment map of the annotation analysis was generated by Cytoscape v3.4.0 to visualize the results.

### PPI network construction analysis

The protein-to-protein (PPI) network was created by using the STRING software v10.0 (http://www.string-db.org) and was drawn to reveal the connection among the overlapping related genes. The amount of nodes and edges were used to identify the most essential related genes for CTD-2547G23.4 in HCC. Hub genes were identified according to the numerical digit of the degrees of each of the nodes and edges. A P value less than 0.05 was regarded to be statistically significant. Pearson’s correlation coefficient was utilized to disclose the relationship between CTD-2547G23.4 and the expression level of hub genes.

## Results

### Differentially expressed lncRNAs in HCC

**Fig. 1:**
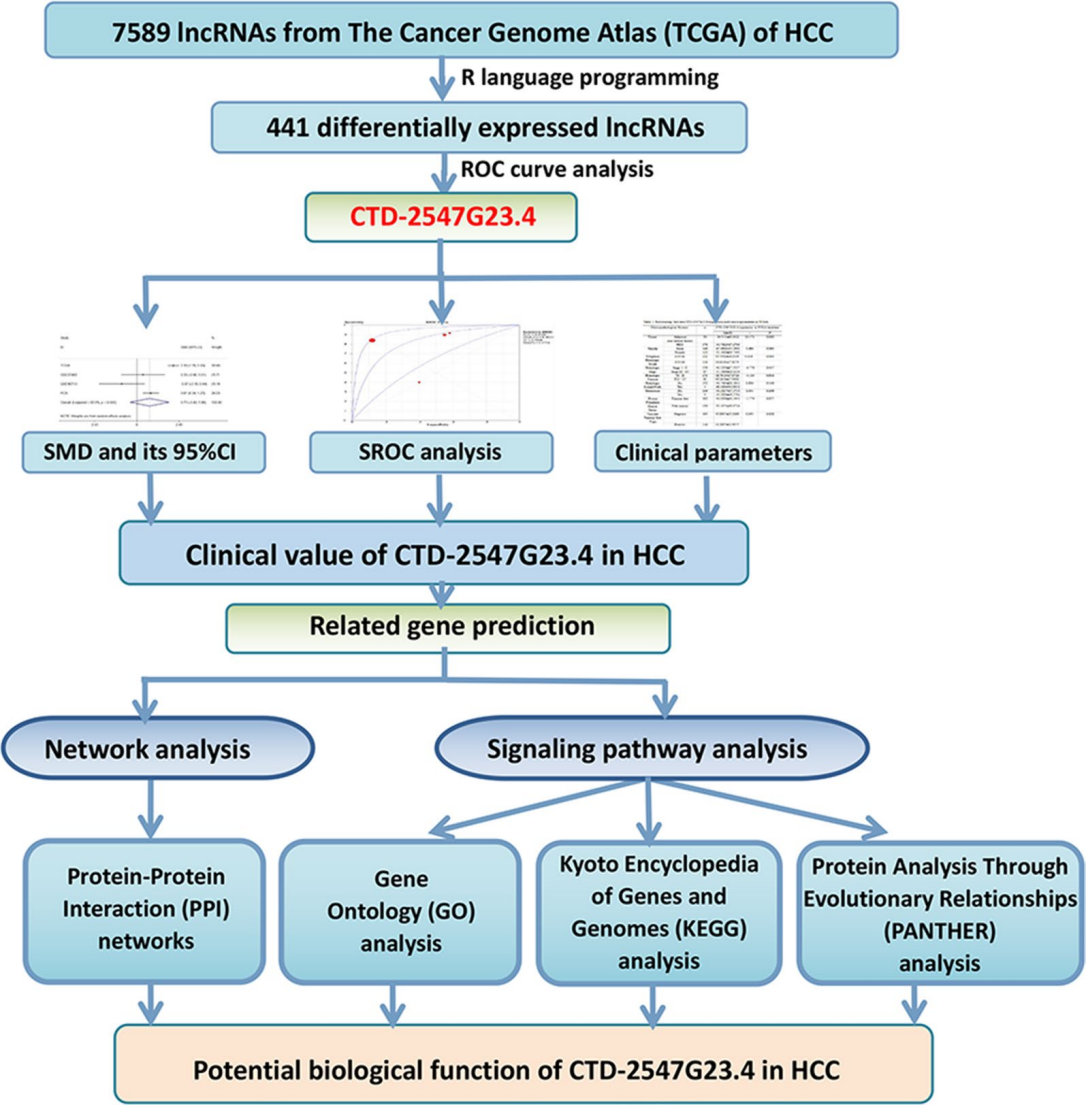
Flow chart of the study procedures in this investigation

**Fig. 2:**
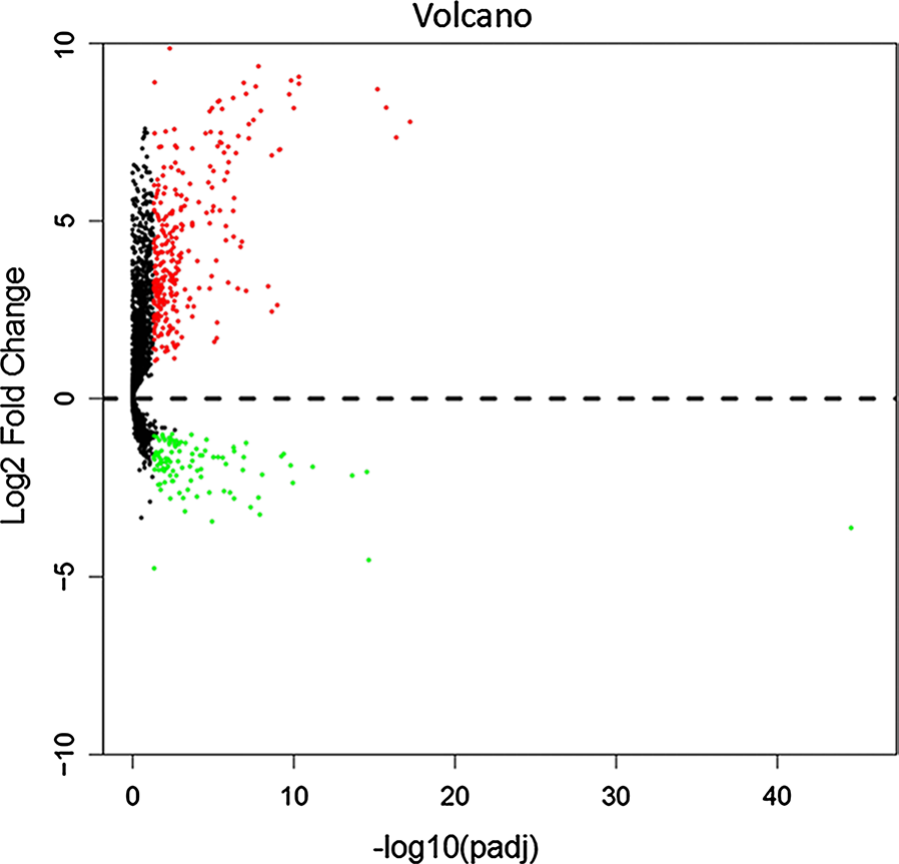
Volcano plot of the differentially expressed lncRNAs between HCC and adjacent non-tumour tissues. The volcano plot was created with the ggplot2 package of R language. The X axis indicates a log2 (fold change), and the Y axis indicates an − log10 (P value). Red represents high expression and green low expression. Black shows the lncRNA expression with both the logFC < 1 and − log10 (P value) < 0.05. Differentially expressed lncRNAs were calculated by DESeqR with 334 overexpressed lncRNAs and 107 underexpressed lncRNAs

Among all the available 60,244 mRNAs expression data downloaded from TCGA portal, 7589 lncRNA were involved. After calculation mentioned above, 441 significantly differentially expressed lncRNAs (334 lncRNAs up-regulated and 107 lncRNAs down-regulated) that met the criteria of log_2_FC > 1 and Padj < 0.05 were obtained (Figs. 1, 2). Subsequently, the diagnostic value of all 441 lncRNAs were analysed by ROC curve analyses. Among them, 46 lncRNAs with AUC values more than 0.900 (data not shown). We conducted the literature research on these lncRNAs and noticed that no investigation were reported concerning part of lncRNAs. CTD-2547G23.4 was finally randomly selected due to its significantly differential expression and unknown biological possesses according to literature.

### The crucial role of CTD-2547G23.4 in the occurrence and progression of HCC evidenced by multiple databases

#### The expression level of CTD-2547G23.4 in HCC via various databases

**Fig. 3:**
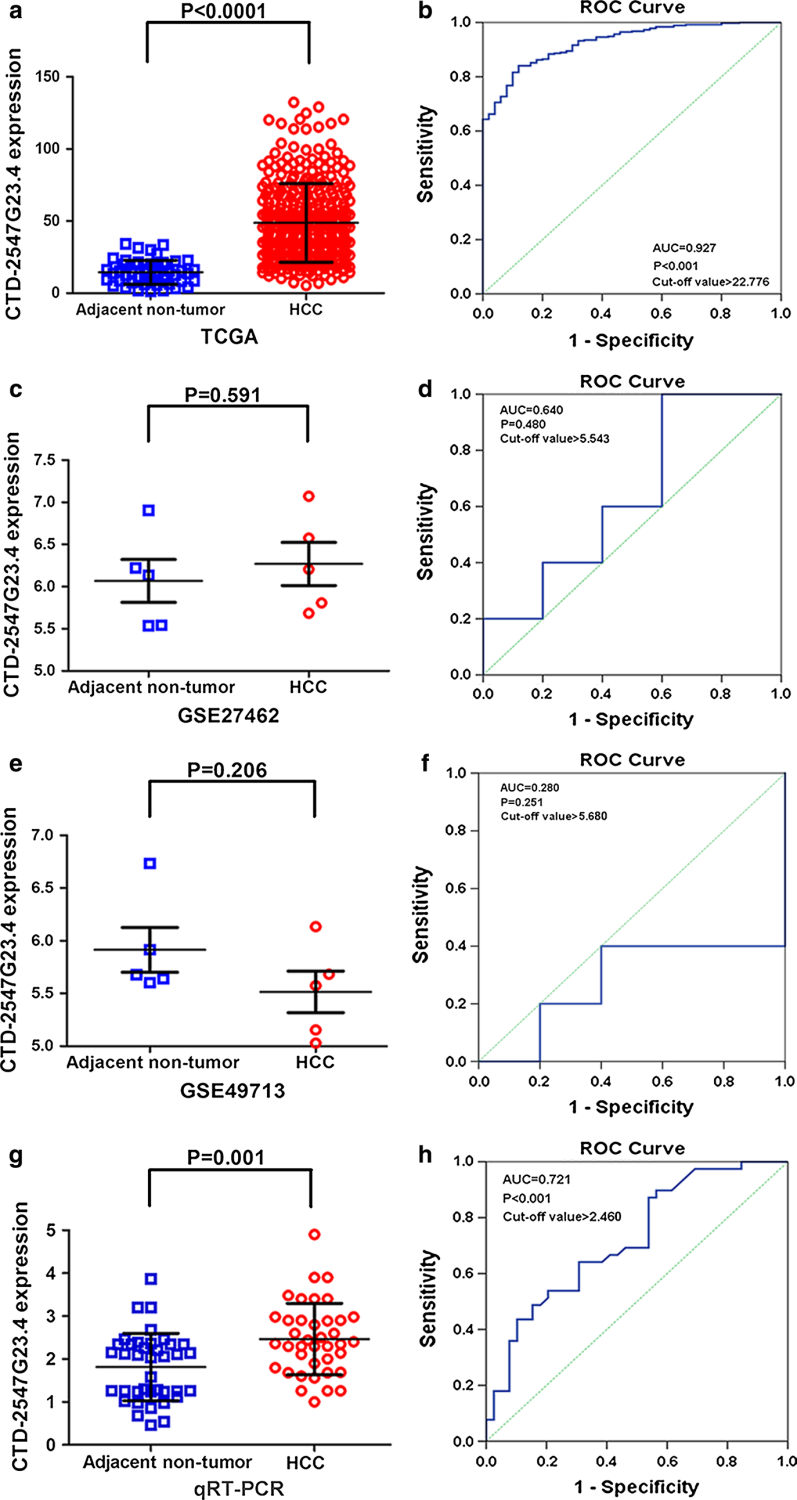
Relative level of CTD-2547G23.4 expression in HCC and adjacent non-cancerous tissues. **a** TCGA data were used to measure CTD-2547G23.4 expression in HCC tissues and matched adjacent non-tumour tissues. **b** Significance of CTD-2547G23.4 in HCC with the ROC curve analysis from TCGA. **c** CTD-2547G23.4 expression value in HCC tissues and adjacent non-tumour tissues from GSE27462. **d** ROC curve of CTD-2547G23.4 in HCC from GSE27462. **e** CTD-2547G23.4 expression level in HCC tissues and adjacent non-tumour tissues from GSE49713. **f** ROC curve of CTD-2547G23.4 in HCC from GSE49713. **g** qRT-PCR was used to measure CTD-2547G23.4 expression in HCC tissues and matched adjacent non-tumour tissues. **h** Diagnostic value of CTD-2547G23.4 in HCC with the ROC curve analysis by qRT-PCR analysis

We first assessed the extracted CTD-2547G23.4 data from TCGA database. The data indicated that CTD-2547G23.4 expression was higher in the 370 HCC samples (48.762 ± 27.270) than in adjacent non-tumour tissues (14.511 ± 8.341) (Fold change = 3.370, P < 0.001; Table 1, Fig. 3a). Subsequently, in other online databases (GEO, ArrayExpress and Oncomine databases), only two chip data (GSE27462 and GSE49713) were obtained from the GEO database, which provided CTD-2547G23.4 expression value in HCC tissues and adjacent non-tumour tissues (Fig. 3c, e). However, the expression of CTD-2547G23.4 did not differ significantly between HCC and adjacent non-tumour tissues in either microarrays. We also examined the relative expression of CTD-2547G23.4 in 39 pairs of HCC tissues matched with adjacent non-tumour tissues by qRT-PCR analysis normalized to GAPDH. The expression level of CTD-2547G23.4 was 2.464 ± 0.833 in HCC tissues, which was predominantly elevated than that in the adjacent non-cancerous tissues (1.813 ± 0.784) (P = 0.001, Table 2, Fig. 3g). To draw a comprehensive conclusion, we integrated the data from TCGA, GEO and in-house PCR using a meta-analysis (Additional file 1: Figure S1). The pooled SMD of CTD-2547G23.4 was 0.730 (95% CI − 0.400 to 1.860, P = 0.206; I^2^ = 92.0%, P < 0.001, Additional file 2: Figure S2A) by the random-effects model. Then, we omitted the GSE27462 and GSE49713 datasets due to small sample sizes, the overall result demonstrated that CTD-2547G23.4 was remarkably up-regulated in HCC (SMD = 1.470, 95% CI 0.190–2.740, P = 0.024; I^2^ = 95.0%, P < 0.001, Additional file 2: Figure S2C). Collectively, the above results certified that CTD-2547G23.4 was evidently elevated in HCC.

**Table 1:** Relationship between CTD-2547G23.4 expression and clinical parameters in TCGA

Clinicopathological feature	n	CTD-2547G23.4 expression in TCGA database		
		M ± SD	t	P
Tissue				
Adjacent non-tumour tissues	50	14.5111 ± 8.3412	18.571	0.000
HCC	370	48.7622 ± 27.2703		
Gender				
Male	248	47.0800 ± 25.2880	1.696	0.091
Female	122	52.1820 ± 30.7392		
Neoplasm histologic grade				
G1 + G2	232	45.7392 ± 26.5334	− 3.028	0.003
G3 + G4	134	54.6165 ± 27.8375		
Pathologic stage				
Stage I–II	259	48.5259 ± 27.5527	− 0.779	0.437
Stage III–IV	87	51.1969 ± 28.0238		
Pathologic tumour				
TI–II	273	48.7915 ± 27.3716	− 0.114	0.910
TIII–IV	93	49.1656 ± 27.4492		
Pathologic lymph node				
No	252	48.7484 ± 28.3941	0.896	0.148
Yes	4	69.5526 ± 31.8511		
Metastasis				
No	266	48.5517 ± 27.1714	1.041	0.299
Yes	4	34.3420 ± 19.3781		
Person neoplasm cancer status				
Tumour free	202	46.8598 ± 26.3441	− 1.774	0.077
With tumour	150	52.1074 ± 28.8736		
Vascular tumour cell type				
Negative	205	45.8987 ± 25.3009	0.045	0.028
Positive	110	53.5897 ± 31.4477

**Table 2:** Relationship between CTD-2547G23.4 expression and clinical parameters in qRT-PCR

Clinicopathological feature	n	CTD-2547G23.4 relevant expression in qRT-PCR		
		M ± SD	t	P
Tissue				
Adjacent non-tumour tissues	39	1.8133 ± 0.7835	3.556^a^	0.001
HCC	39	2.4646 ± 0.8332		
Gender				
Male	29	2.4124 ± 0.8563	− 0.661	0.512
Female	10	2.6160 ± 0.7845		
Age (years)				
< 50	18	2.2156 ± 0.8285	1.777	0.084
≥ 50	21	2.6781 ± 0.7949		
Pathologic tumour				
TI–II	25	2.4428 ± 0.7728	− 0.216	0.830
TIII–IV	14	2.5036 ± 0.9612		
Nodes				
Single	34	2.4444 ± 0.8282	− 0.390	0.698
Multi	5	2.6020 ± 0.9537		
Metastasis				
No	36	2.3906 ± 0.6916	− 0.882	0.469
Yes	3	3.3533 ± 1.88057		
Embolus				
No	38	2.4821 ± 0.8371	0.804	0.426
Yes	1	1.8000		
Status				
Alive	17	2.4688 ± 0.9286	0.225	0.824
Death	7	2.5586 ± 0.7667		
Diameter (cm)				
< 5	11	2.3573 ± 0.7985	− 0.499	0.621
≥ 5	28	2.5068 ± 0.8569		
Vascular infiltration				
No	33	2.4570 ± 0.7447	− 0.133	0.895
Yes	6	2.5067 ± 1.3130		
Tumor capsular infiltration				
Infiltration or no capsule	14	2.3057 ± 0.8561	0.889	0.380
With complete capsule	25	2.5536 ± 0.8241		
Differentiation				
Low	10	2.2240 ± 0.5027	F = 2.047^b^	0.144
Moderate	25	2.4448 ± 0.8506		
High	4	3.1900 ± 1.1612		
AFP				
No	21	2.5957 ± 1.0130	− 0.619	0.541
Yes	12	2.4192 ± 0.6248		
Cirrhosis				
No	25	2.6456 ± 0.8324	− 1.872	0.069
Yes	14	2.1414 ± 0.7580		

^a^ Student’s paired t test

^b^ One-way analysis of variance (ANOVA) test

#### Further verification of CTD-2547G23.4 up-regulation in HCC by SROC

**Fig. 4:**
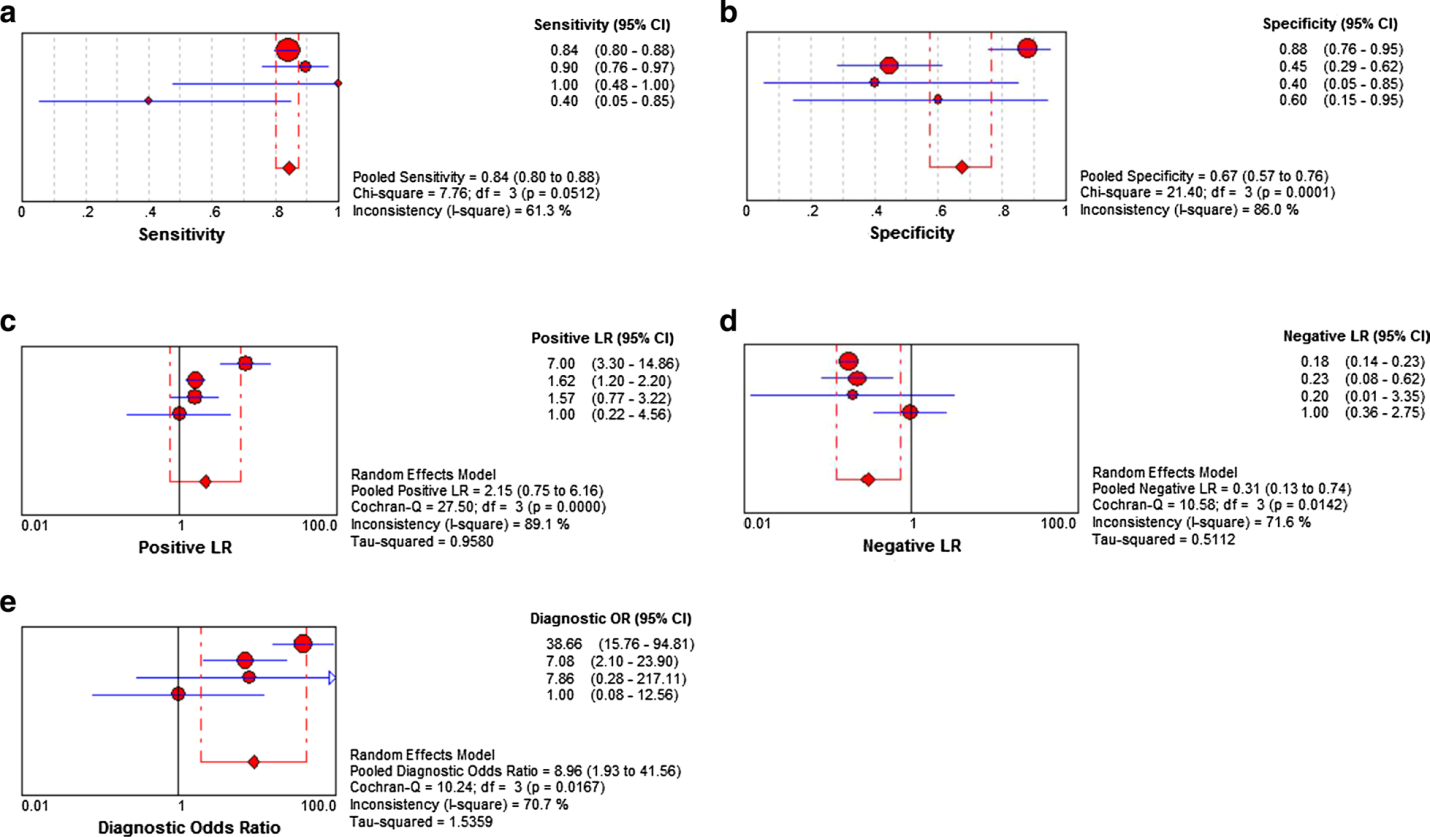
Forest plots exhibit diagnostic performance of CTD-2547G23.4 in HCC. **a** The pooled sensitivity. **b** The pooled specificity. **c** The pooled positive likelihood ratio. **d** The pooled negative likelihood ratio. **e** The pooled diagnostic odds ratio based upon the eligible datasets

To further identify the capability of CTD-2547G23.4 in distinguishing cancer from non-cancerous liver tissues, ROC and SROC curve analyses were carried out. The AUC of CTD-2547G23.4 from TCGA data was 0.927 (P < 0.001, cut-off > 22.776, Fig. 3b). The AUC of CTD-2547G23.4 from GSE27462 and GSE49713 were 0.640 (P = 0.480, cut-off > 5.543, Fig. 3d) and 0.280 (P = 0.251, cut-off > 5.680, Fig. 3f) respectively. The AUC of CTD-2547G23.4 from the 39 HCC patients was 0.721 (P < 0.001, cut-off > 2.460, Fig. 3h). From meta-analysis, the AUC of SROC was 0.816 (95% CI 0.654–0.976) (Additional file 2: Figure S2B). The pooled sensitivity, specificity, positive likelihood ratio (PLR), negative likelihood ratio (NLR), diagnostic odds ratio (DOR) of CTD-2547G23.4 in these studies were 0.840 (95% CI 0.800–0.880), 0.67 (95% CI 0.570–0.760), 2.15 (95% CI 0.750–6.160), 0.31 (95% CI 0.130–0.740) and 8.96 (95% CI 1.930–41.560), respectively (Fig. 4a–e). Then, we omitted the GSE49713 dataset due to its low capability to distinguish the cancer from para-cancerous tissues. The AUC of SROC was 0.8690 (95% CI 0.758–0.980) (Additional file 2: Figure S2D). Based on the described above, CTD-2547G23.4 was up-regulated in HCC.

### Clinical implication of CTD-2547G23.4 in the progression of HCC

Considering the potential oncogenic role of CTD-2547G23.4 in the progression of HCC, we analysed the relationship between clinicopathological. Significantly different expression values of CTD-2547G23.4 were observed between high and low neoplasm histologic grades. The obvious difference also occurred between negative and positive vascular tumour cell types (Table 1). Regarding neoplasm histologic grade, CTD-2547G23.4 expression levels were overexpressed in samples with GIII + GIV vs. GI + GII (54.617 ± 27.838 vs. 45.739 ± 26.533, P = 0.003, Table 1). Samples with vascular tumour cell type versus without vascular tumour cell type had up-regulated CTD-2547G23.4 level (53.590 ± 31.448 vs. 45.899 ± 25.301, P = 0.028, Table 1). However, expression of CTD-2547G23.4 did not significantly correlate with other clinicopathological features. Other online databases did not provide specific clinicopathological parameters data. Simultaneously, we also investigated relationship between CTD-2547G23.4 expression and clinicopathological significance using PCR data. No significant difference concerning the CTD-2547G23.4 expression was detected between different groups of clinical parameters (Table 2), which could be probably due to insufficient size of cases we collected.

### Knockdown of CTD-2547G23.4 arrests the cell cycle of HCC cells

**Fig. 5:**
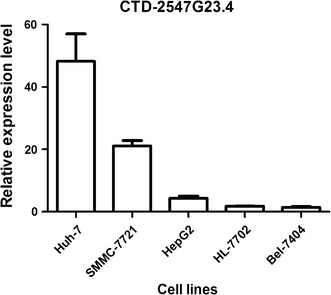
Relative expression level of CTD-2547G23.4 in five HCC cell lines

**Fig. 6:**
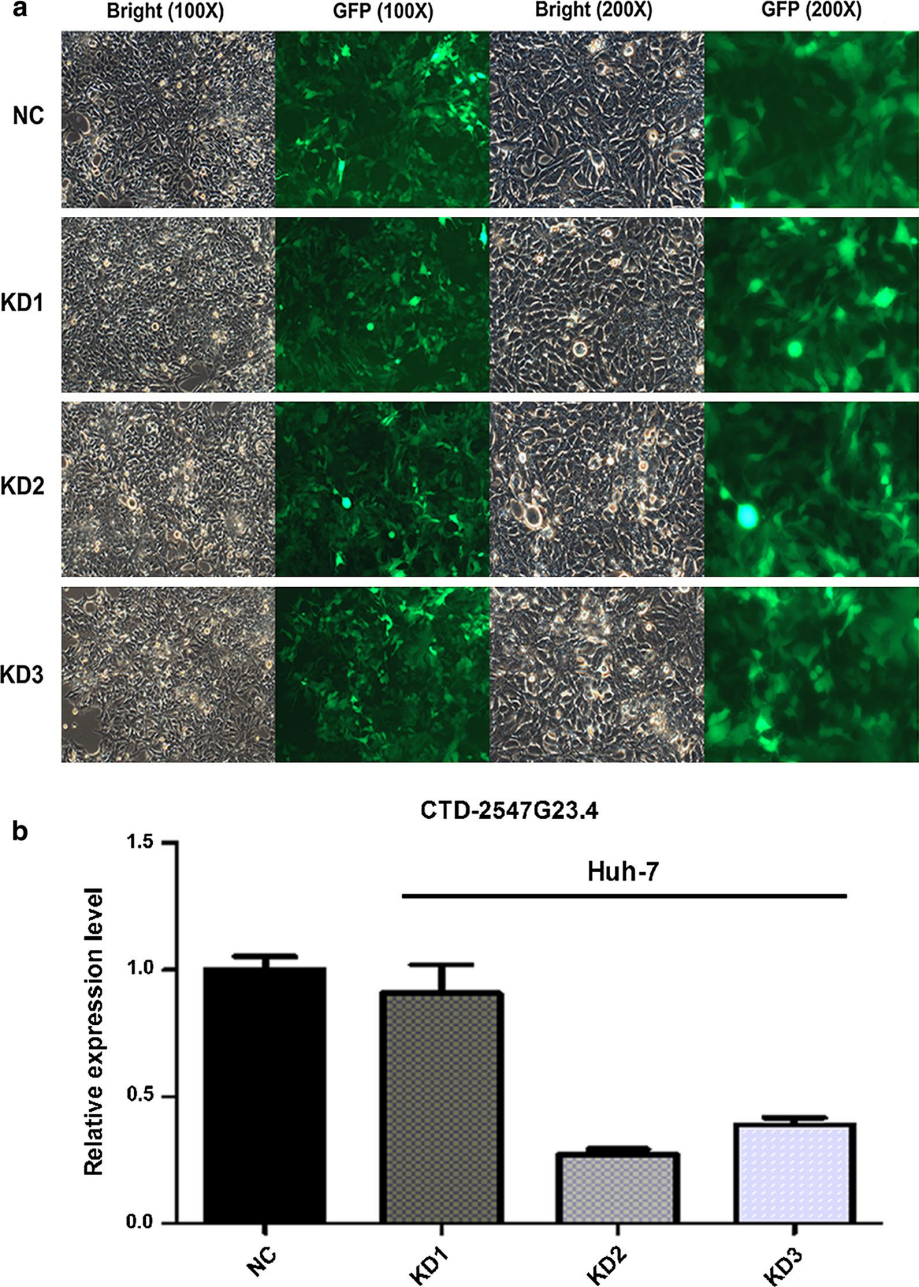
Knockdown (KD) of CTD-2547G23.4 using lentivirus-mediated siRNA in Huh-7 cell line. **a** Microscopic analysis of Huh-7 cells 72 h following infection with lentivirus-mediated siRNA (*NC* negative control, *GFP* green fluorescent protein). **b** qRT-PCR analysis of CTD-2547G23.4 relative expression level in Huh-7 cells

**Fig. 7:**
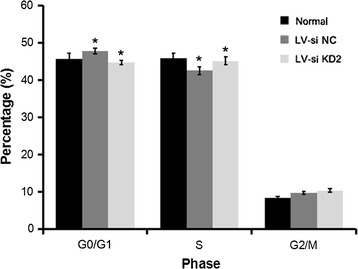
Percentage of cells in G0/G1, S and G2/M phase of the cell cycle (*P < 0.05)

To explore the potential role of CTD-2547G23.4 in HCC. We firstly detected the expression level of CTD-2547G23.4 in five HCC cell lines (Huh-7, SMMC-7721, HepG2, BEL-7404 and HL-7702) by qRT-PCR and discovered that CTD-2547G23.4 was widely expressed and subsequently chose Huh-7 cell line for the following investigation due to the highest expression level among five HCC cell lines (Fig. 5). To further probe the role of CTD-2547G23.4 in HCC cell cycle, lentivirus-mediated small interfering RNA (siRNA) was applied to silence CTD-2547G23.4 expression in Huh-7 cell line. The relative expression of CTD-2547G23.4 was significantly downregulated in CTD-2547G23.4 knockdown (KD) 2 group of Huh-7 cell line (0.270 ± 0.038 vs. 1.000 ± 0.087, P < 0.001, Fig. 6). Moreover, cell cycle analysis revealed that CTD-2547G23.4 depletion in Huh-7 cell line led to S phase arrest (Fig. 7).

### Related genes of CTD-2547G23.4 and gene-annotation enrichment analysis

**Fig. 8:**
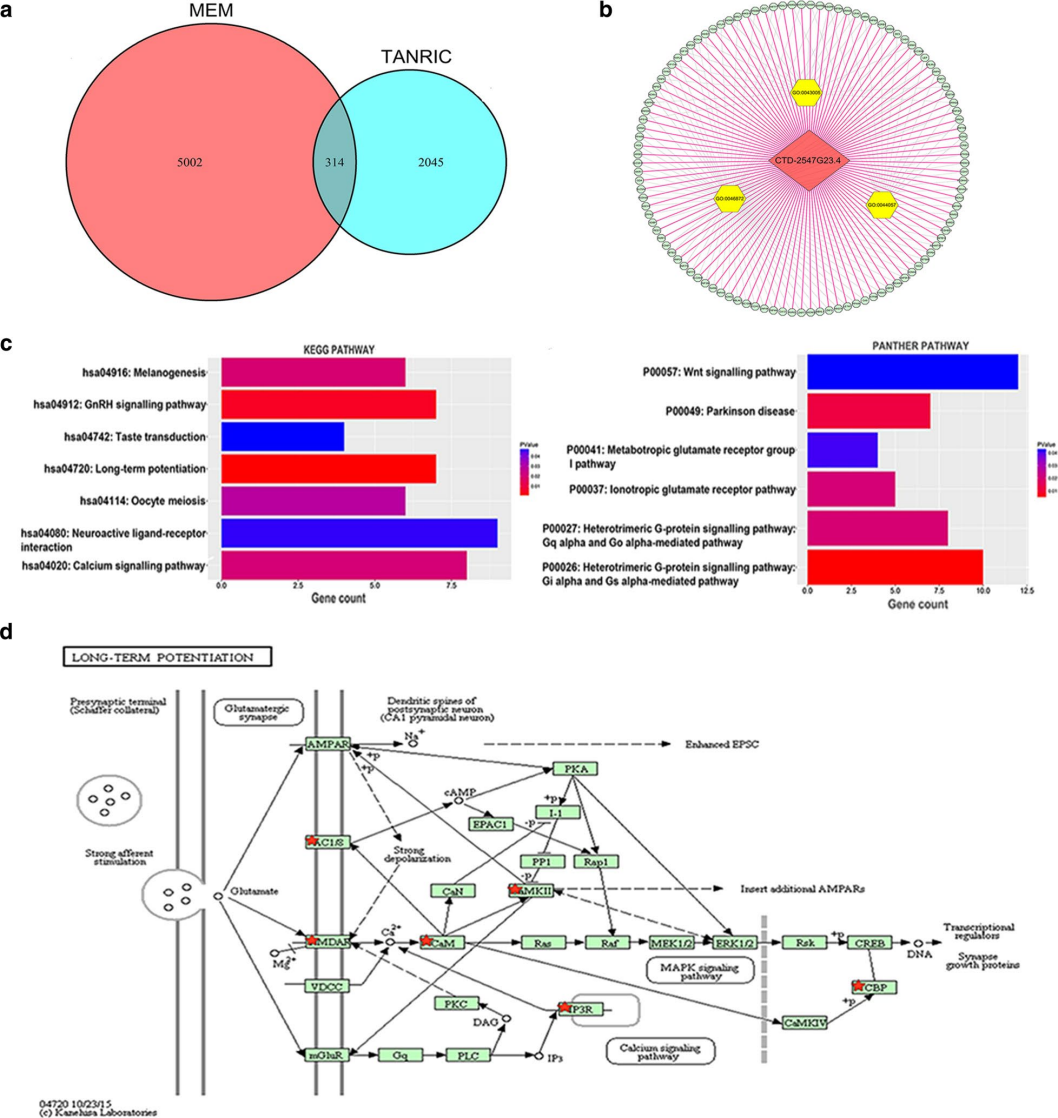
The enriched annotation pathways analysis of potential genes targeted by CTD-2547G23.4 in HCC. **a** Venn diagram of the overlap between the number of predicted target genes using the MEM and TANRIC databases. **b** The significantly enriched annotation of the Gene Ontology (GO) categories. **c** The significantly enriched annotation of the KEGG and PANTHER pathway. **d** KEGG pathway map illustrating the long-term potentiation signalling pathway in humans by DAVID 6.8 (https://david.ncifcrf.gov/)

The online TANRIC software collected 2045 related genes, and we identified more than 5002 genes from the MEM database. We eventually identified 314 overlapping genes (Fig. 8a) and the DAVID analysis was implemented to identify GO annotations and KEGG and PANTHER pathways. “Regulation of system process” was the most significantly enriched biological process (BP) (P = 0.002, Table 3). According to the cellular component (CC) analysis, genes mostly assembled at the neuronal projections (P < 0.001, Table 3). The genes from GO molecular functions (MFs) were enriched in metal ion binding (P < 0.001, Table 3). The three most significantly enriched annotations of GO categories were GO:0043005, GO:0044057 and GO:0046872 (Fig. 8b). In addition, the related genes of CTD-2547G23.4 in the KEGG enrichment analysis were shown to be particularly related to long-term potentiation (P < 0.001, Table 4, Fig. 8c) with seven genes (ADCY8, GRIN2C, CREBBP, GRIN2A, CALML5, ITPR3, CAMK2A) (Fig. 8d). The PANTHER pathway was mainly enriched in the heterotrimeric G-protein signalling pathway (P = 0.004, Table 4).

**Table 3:** Gene Ontology (GO) analysis of the potential targets of CTD-2547G23.4

Category	Term	Count	P value
GOTERM_BP	GO:0044057—regulation of system process	14	1.72E−03
GOTERM_BP	GO:0019226—transmission of nerve impulse	14	5.05E−03
GOTERM_BP	GO:0060078—regulation of postsynaptic membrane potential	4	5.98E−03
GOTERM_BP	GO:0045449—regulation of transcription	59	6.20E−03
GOTERM_BP	GO:0032095—regulation of response to food	3	6.97E−03
GOTERM_BP	GO:0032098—regulation of appetite	3	6.97E−03
GOTERM_BP	GO:0007267—cell–cell signalling	19	9.61E−03
GOTERM_BP	GO:0007268—synaptic transmission	12	9.95E−03
GOTERM_BP	GO:0030182—neuron differentiation	15	1.30E−02
GOTERM_BP	GO:0006350—transcription	48	1.37E−02
GOTERM_CC	GO:0043005—neuron projection	16	2.28E−04
GOTERM_CC	GO:0044459—plasma membrane part	54	2.76E−04
GOTERM_CC	GO:0045202—synapse	16	3.39E−04
GOTERM_CC	GO:0030054—cell junction	18	2.32E−03
GOTERM_CC	GO:0005886—plasma membrane	76	3.83E−03
GOTERM_CC	GO:0042734—presynaptic membrane	4	9.41E−03
GOTERM_CC	GO:0042995—cell projection	20	9.66E−03
GOTERM_CC	GO:0008076—voltage-gated potassium channel complex	6	9.88E−03
GOTERM_CC	GO:0034705—potassium channel complex	6	9.88E−03
GOTERM_CC	GO:0044456—synapse part	10	1.28E−02
GOTERM_MF	GO:0046872—metal ion binding	92	3.60E−04
GOTERM_MF	GO:0043169—cation binding	92	5.10E−04
GOTERM_MF	GO:0043167—ion binding	92	8.69E−04
GOTERM_MF	GO:0008066—glutamate receptor activity	5	1.51E−03
GOTERM_MF	GO:0008270—zinc ion binding	55	2.74E−03
GOTERM_MF	GO:0035254—glutamate receptor binding	3	6.90E−03
GOTERM_MF	GO:0022843—voltage-gated cation channel activity	8	1.02E−02
GOTERM_MF	GO:0005244—voltage-gated ion channel activity	9	1.44E−02
GOTERM_MF	GO:0022832—voltage-gated channel activity	9	1.44E−02
GOTERM_MF	GO:0005261—cation channel activity	11	1.45E−02

**Table 4:** KEGG and PANTHER pathway analyses of the validated targets of CTD-2547G23.4

Category	Term	Count	P value
KEGG_PATHWAY	hsa04720: long-term potentiation	7	6.32E−04
KEGG_PATHWAY	hsa04912: GnRH signalling pathway	7	4.18E−03
KEGG_PATHWAY	hsa04916: melanogenesis	6	1.92E−02
KEGG_PATHWAY	hsa04020: calcium signalling pathway	8	2.00E−02
KEGG_PATHWAY	hsa04114: Oocyte meiosis	6	2.88E−02
KEGG_PATHWAY	hsa04080: neuroactive ligand-receptor interaction	9	4.68E−02
KEGG_PATHWAY	hsa04742: taste transduction	4	4.78E−02
PANTHER_PATHWAY	P00026: heterotrimeric G-protein signalling pathway: Gi alpha and Gs alpha-mediated pathway	10	4.43E−03
PANTHER_PATHWAY	P00049: parkinson disease	7	1.15E−02
PANTHER_PATHWAY	P00027: heterotrimeric G-protein signalling pathway: Gq alpha and Go alpha-mediated pathway	8	1.98E−02
PANTHER_PATHWAY	P00037: Ionotropic glutamate receptor pathway	5	2.03E−02
PANTHER_PATHWAY	P00041: metabotropic glutamate receptor group I pathway	4	4.10E−02
PANTHER_PATHWAY	P00057: Wnt signalling pathway	12	4.25E−02

### PPI network construction and module analysis

**Fig. 9:**
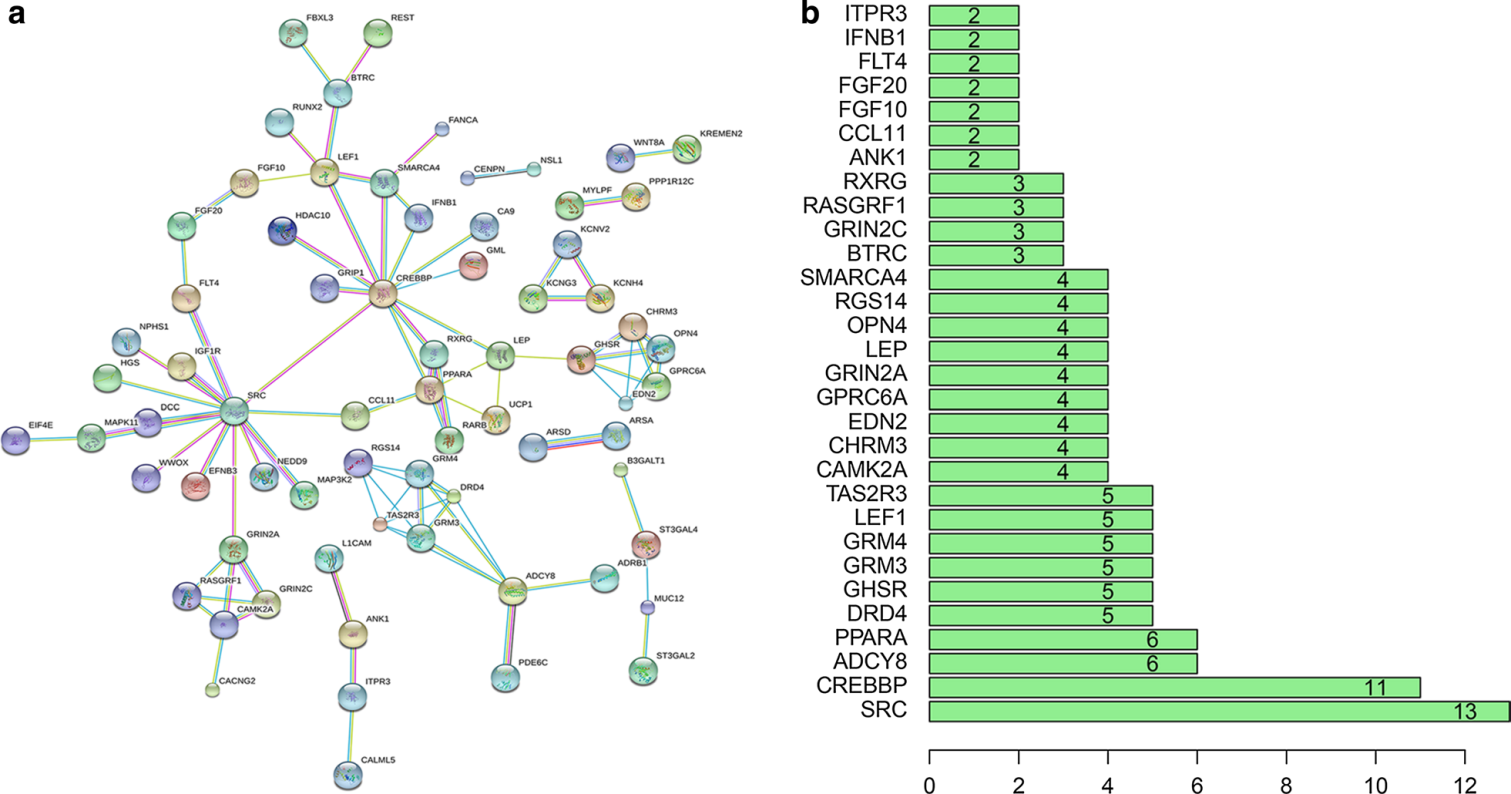
Protein–protein interaction (PPI) network analysis. **a** PPI network of CTD-2547G23.4 target genes in HCC. **b** The 30 most significant potential hub genes targeted by CTD-2547G23.4 in HCC

**Fig. 10:**
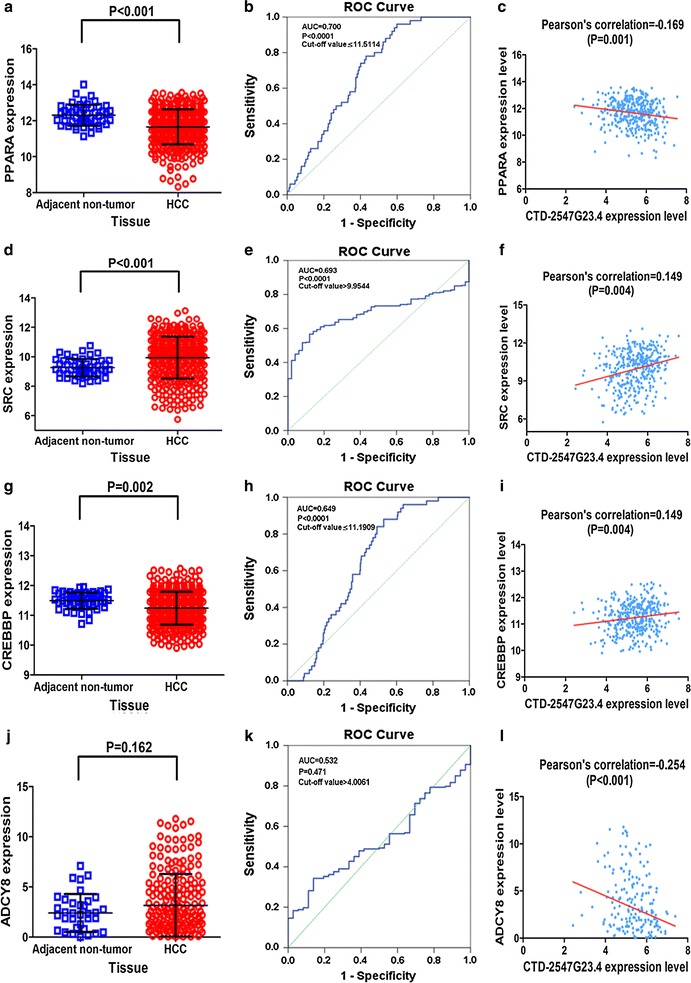
Relative expression level of 4 hub genes in HCC and adjacent non-cancerous tissues from TCGA. **a** PPARA, **d** SRC, **g** CREBBP, **j** ADCY8. Diagnostic value of CTD-2547G23.4 in HCC with the ROC curve analysis. **b** PPARA, **e** SRC, **h** CREBBP, **k** ADCY8. Correlation analysis between CTD-2547G23.4 and hub genes through Pearson’s correlation. **c** PPARA, **f** SRC, **i** CREBBP, **l** ADCY8

The PPI network contained 301 nodes and 88 edges (Fig. 9a). Among these genes, the degree values of more than 2 were defined as being indicative of hub genes (Fig. 9b). Sarcoma (SRC, degree = 13), cyclic AMP responsive element-binding protein (CREBBP, degree = 11), adenylate cyclase 8 (ADCY8, degree = 6) and peroxisome proliferator activated receptor alpha (PPARA, degree = 6) were the four most symbolic hub genes. We found that the PPARA and CREBBP expression value in HCC tissues was clearly down-regulated than in matched non-tumour tissues (P < 0.01, Fig. 10). The SRC expression level in HCC tissues was obviously up-regulated than in matched non-tumour tissues (P < 0.001, Fig. 10). The AUC values of PPARA, SRC, CREBBP and ADCY8 were 0.700 (P < 0.001), 0.693 (P < 0.001), 0.649 (P < 0.001) and 0.532 (P = 0.471), respectively. Through Pearson’s correlation analysis, PPARA (r = − 0.169, P = 0.001), SRC (r = 0.149, P = 0.004), CREBBP (r = 0.149, P = 0.004), and ADCY8 (r = − 0.254, P < 0.001) were significantly related to CTD-2547G23.4 in TCGA (Fig. 10).

## Discussion

The current study, to the best of our knowledge, was the first to investigate a novel lncRNA CTD-2547G23.4 in HCC, which was significantly up-regulated in HCC samples. We found that increased CTD-2547G23.4 expression was associated with the neoplasm histologic grade and vascular tumour cell type, and these clinic pathological characteristics could be reprehensive of poor outcome. In addition, SROC curve analysis demonstrated that CTD-2547G23.4 provided high diagnostic performance for the detection of HCC, with an AUC 0.8156 based on TCGA, GSE27462, GSE49713 and qRT-PCR data. Moreover, we also carried out a sequential in silico prediction for the related genes of CTD-2547G23.4 in HCC and observed that CTD-2547G23.4 targeted hub genes related to the tumourigenesis and development of HCC. Based on these findings, we proposed that CTD-2547G23.4 may be a candidate clinical index and exert an oncogenic role in the progression of HCC.

To date, an accumulating number of investigations have identified that aberrant lncRNA expression levels often play a crucial role in the biological process of HCC. For instance, the lncRNA HULC, which is highly up-regulated in HCC, should serve as a scaffold for ERK and YB-1 to enhance hepatocarcinogenesis [20]. Liu J et al. [21] determined that the lncRNA SNHG20 was significantly overexpressed in HCC tissues. Interestingly, its high expression level leads to EMT-induced HCC cell invasion via binding to the enhancer of EZH2. HOTAIR is a long non-coding RNA that is overexpressed in HCC tissues and drives HCC cell proliferation and progression [22]. These findings could also afford new insight into the molecular biological mechanisms of HCC that depend on lncRNAs.

More importantly, HCC is not frequently diagnosed until the late stage. Therefore, the identification of a suitable biomarker with good diagnostic performance is urgent. Alpha-fetoprotein (AFP) has been used as the most common clinical screening and diagnosis method for HCC. AFP has been reported to have a sensitivity from 39 to 64% and specificity from 76 to 91% [23]. The suboptimal sensitivity and specificity of AFP are probably due to a variety of factors. Recently, the versatile diagnostic role of lncRNAs in various cancer types, including HCC, has attracted the attention of many scholars. A meta-analysis of ten studies with 820 HCC patients and 785 healthy controls determined that lncRNAs had a high diagnostic significance for HCC, and their expression could theoretically be used as auxiliary biomarkers for confirmation of HCC [24]. For example, the overexpression of the lncRNA HULC in HCC could act as a promising biomarker for detecting and screening hepatocarcinogenesis [25, 26]. All of these studies suggest that lncRNA CTD-2547G23.4 may serve as a predictor for HCC diagnosis. Herein, we carried out SMD and SROC curve analyses. Simultaneously, the heterogeneity between the AUC of the two cohorts may be owing to the different methods used for the evaluation of the expression levels of CTD-2547G23.4 and the different number of samples.

We have demonstrated that CTD-2547G23.4 is overexpressed in HCC and might have potential diagnostic value for HCC patients. To investigate the clinical value of CTD-2547G23.4 in HCC diagnosis and prognosis, we analysed the relationship between the expression of CTD-2547G23.4 and clinic pathological characteristics. Surprisingly, through analysing the expression data from TCGA datasets, we observed that elevated expression of CTD-2547G23.4 was significantly related to a high neoplasm histologic grade and vascular tumour cell type. The histologic grade often reflects the tumour growth and invasion rate and predicts the clinical outcome [27]. Vascular tumour cell type is also closely related to the growth of tumours. These observations suggest that against CTD-2547G23.4 might be more effective in tumours of high histological grade and effective after resection in the prevention of recurrences. Furthermore, the varying degrees of the CTD-2547G23.4 expression may limit the effect of various types of therapy.

Protein–protein interaction network evaluation assisted to identify 30 hub genes that were the core related genes of CTD-2547G23.4 in HCC. Among these 30 hub genes, SRC, CREBBP, ADCY8, and PPARA were the four core genes. We next focused on the roles and functions of SRC, CREBBP and PPARA in HCC for deepgoing discussion.

The role of SRC, as the proto-oncogene encoding a tyrosine kinase, has been studied in multiple tumours for many years [28, 29]. Similarly, many studies have indicated that SRC is involved in various signalling pathways of HCC [30, 31]. Zhao et al. [32] demonstrated that SRC was markedly elevated in HCC tissues and related to clinical stage, pathological differentiation, and the status of lymph node metastasis. In addition, SRC was discovered to enhance HCC cell invasion and metastasis via phosphorylating the EGFR pathway [33]. Moreover, the underlying mechanism of EF24-suppressed invasion and migration of hepatocellular carcinoma has been shown to be through inducing the phosphorylation of SRC [34]. Thus, SRC is highly involved in HCC. But, the association between SRC and CTD-2547G23.4 has not been reported. As we predicted, SRC is the most significant hub gene of CTD-2547G23.4 in HCC. In-depth studies are essential to validate this correlation between SRC and CTD-2547G23.4 in HCC.

CREBBP is a transcriptional co-activator with an essential function in the liver through its regulation of gene expression and diverse processes such as gluconeogenesis, lipid metabolism, and cell proliferation [35]. Lately, some studies have shown that CREBBP is associated with apoptosis in HCC. Chen et al. [36] found that the CREB pathway was partly involved in tumour apoptosis caused by *N*-butylidenephthalide. Moreover, Abramovitch et al. [37] demonstrated that CREBBP played a central role in the anti-apoptotic effect in HCC through in vitro and in vivo experiments.

The PPARA gene encodes PPAR-alpha, which is a transcription factor that maintains hepatic metabolic homeostasis through the hepatocyte nuclear factor-4 alpha (HNF4A) gene [38]. The abnormal stimulation of PPAR has also been reported to generate HCC. In addition, Drakaki and his colleagues have proven that miR-9 plays key roles in the early stages of HCC oncogenesis through direct regulation of PPARA [39]. Yamasaki investigated cell proliferation in fenofibrate-treated HCC cells and elucidated that fenofibrate induced an antiproliferative effect via a PPAR-alpha-dependent mechanism [40]. These findings suggest that PPARA is vital in HCC and may be a molecular target for therapy. Due to these findings and the results of the PPI network analysis, we put forward a hypothesis that CTD-2547G23.4 modulates the process of HCC via targeting PPARA. Nevertheless, target gene in silico prediction algorithms have limited specificity. Therefore, further in vitro and in vivo assays of CTD-2547G23.4 potential biological function in those signaling pathways in HCC is essential to verify and illuminate the regulative mechanisms of CTD-2547G23.4 in HCC. Further investigations are required to fully elucidate this hypothesis.

## Conclusions

In conclusion, we have demonstrated high lncRNA CTD-2547G23.4 expression in HCC and analysed the related genes and pathways of CTD-2547G23.4 through bioinformatics methods. CTD-2547G23.4 could target hub genes such as SRC, CREBBP and PPARA, which regulate the biological process in HCC. Our study has provided the first demonstration that lncRNA CTD-2547G23.4 could be a useful clinical index and furnished insights into the better understanding of the potential mechanism of CTD-2547G23.4 in HCC.

## Additional files

**Additional file 1: Figure S1.** Flow diagram of the selection in three databases.

**Additional file 2: Figure S2.** The expression level of CTD-2547G23.4 in HCC. (A) Forest plot of all eligible datasets evaluating CTD-2547G23.4 expression between HCC and adjacent non-tumour tissues. (B) The SROC curve for the differentiation of HCC from adjacent non-tumour tissues based upon TCGA, GSE27462, GSE49713 and qRT-PCR datasets. (C) Forest plot of TCGA and in-house PCR datasets evaluating CTD-2547G23.4 expression between HCC and adjacent non-tumour tissues. (D) The SROC curve for the differentiation of HCC from adjacent non-tumour tissues based upon TCGA, GSE27462 and qRT-PCR datasets.

## Abbreviations

HCC: hepatocellular carcinoma
lncRNA: long non-coding RNA
TCGA: The Cancer Genome Atlas
GEO: Gene Expression Omnibus
ROC: receiver operating characteristic
TANRIC: The Atlas of Noncoding RNAs in Cancer
MEM: Multi Experiment Matrix
GO: Gene Ontology
BP: biological process
CC: cellular component
MF: molecular function
KEGG: Kyoto Encyclopedia of Genes and Genomes
PANTHER: Protein Analysis Through Evolutionary Relationships
PPI: protein–protein interaction
