# Supplementary figures and images for: Up-regulation of CTD-2547G23.4 in hepatocellular carcinoma tissues and its prospective molecular regulatory mechanism: a novel qRT-PCR and bioinformatics analysis study
Source: Cancer Cell Int. 2018 May 11;18:74. doi: 10.1186/s12935-018-0566-3 (PMC5948809; doi:10.1186/s12935-018-0566-3)

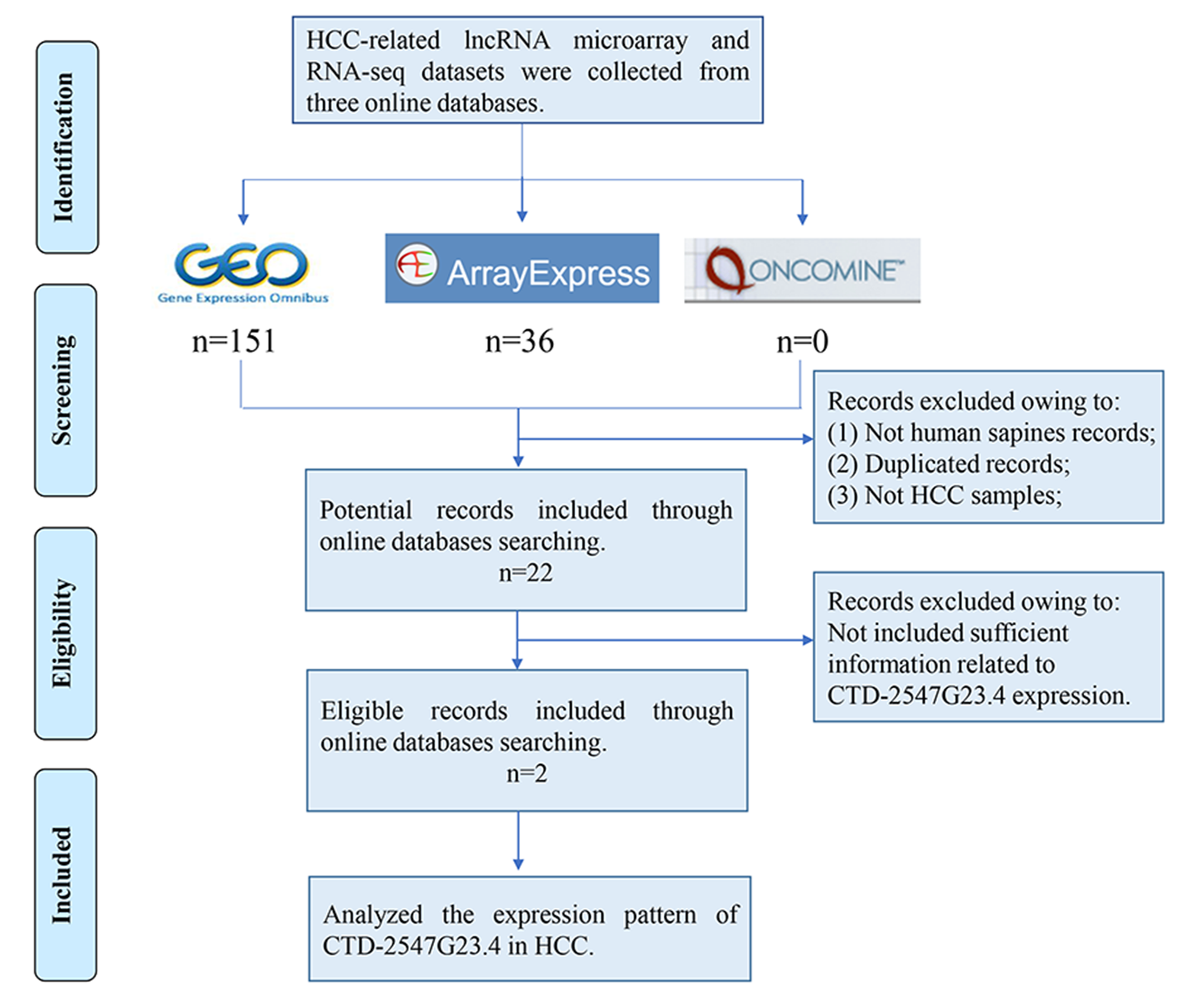

Supplement: Supplementary file 1 — Additional file 1: Figure S1. Flow diagram of the selection in three databases. [file 12935_2018_566_MOESM1_ESM.tif]

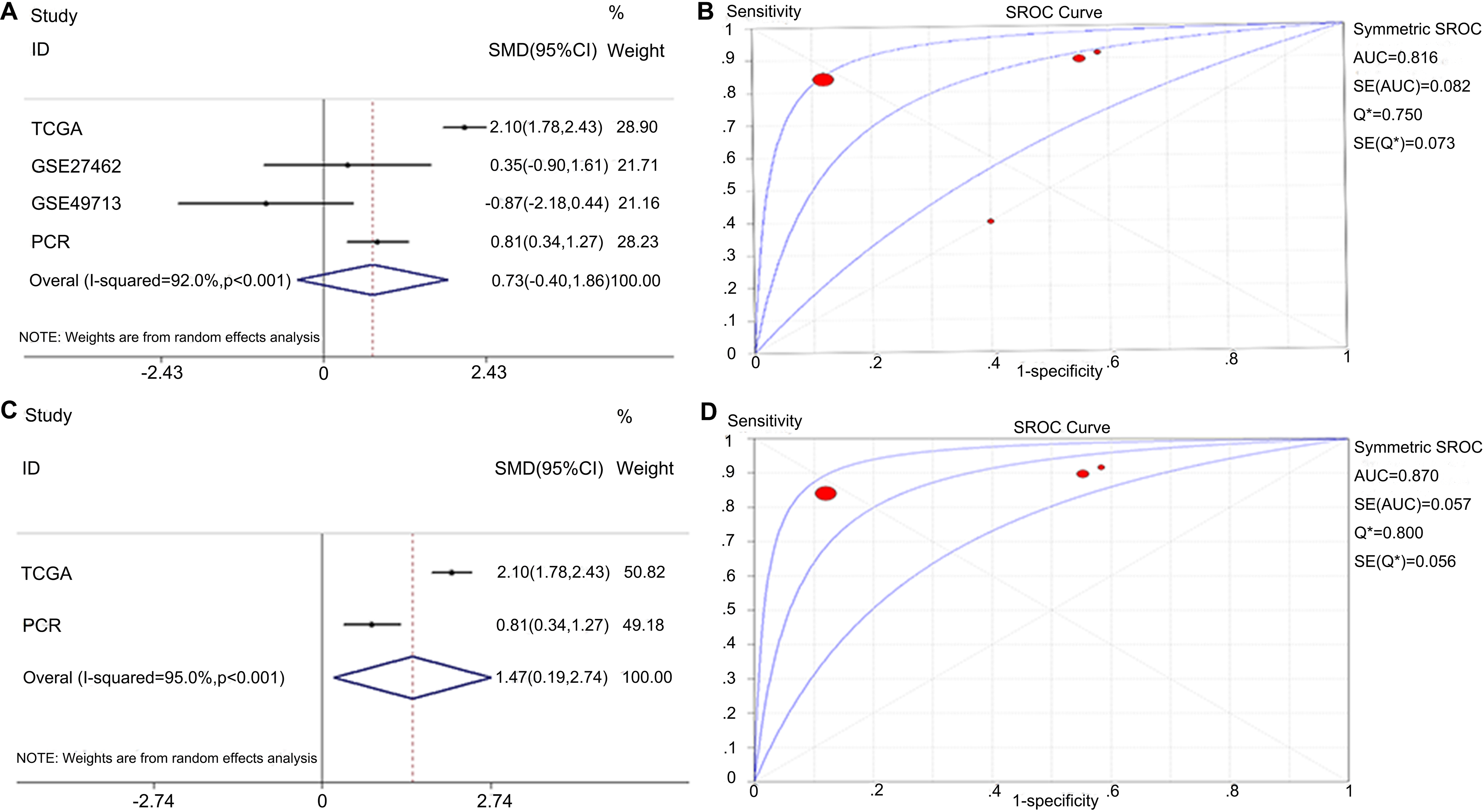

Supplement: Supplementary file 2 — Additional file 2: Figure S2. The expression level of CTD-2547G23.4 in HCC. (A) Forest plot of all eligible datasets evaluating CTD-2547G23.4 expression between HCC and adjacent non-tumour tissues. (B) The SROC curve for the differentiation of HCC from adjacent non-tumour tissues based upon TCGA, GSE27462, GSE49713 and qRT-PCR datasets. (C) Forest plot of TCGA and in-house PCR datasets evaluating CTD-2547G23.4 expression between HCC and adjacent non-tumour tissues. (D) The SROC curve for the differentiation of HCC from adjacent non-tumour tissues based upon TCGA, GSE27462 and qRT-PCR datasets. [file 12935_2018_566_MOESM2_ESM.tif]
